# Manganese-Induced Atypical Parkinsonism Is Associated with Altered Basal Ganglia Activity and Changes in Tissue Levels of Monoamines in the Rat

**DOI:** 10.1371/journal.pone.0098952

**Published:** 2014-06-04

**Authors:** Safa Bouabid, Claire Delaville, Philippe De Deurwaerdère, Nouria Lakhdar-Ghazal, Abdelhamid Benazzouz

**Affiliations:** 1 Univ. de Bordeaux, Institut des Maladies Neurodégénératives, UMR 5293, Bordeaux, France; 2 CNRS, Institut des Maladies Neurodégénératives, UMR 5293, Bordeaux, France; 3 Université Mohamed V-Agdal, Faculté des Sciences, Equipe Rythmes Biologiques, Neurosciences et Environnement, Rabat, Morocco; The Chinese University of Hong Kong, Hong Kong

## Abstract

Manganese neurotoxicity is associated with motor and cognitive disturbances known as Manganism. However, the mechanisms underlying these deficits remain unknown. Here we investigated the effects of manganese intoxication on motor and non-motor parkinsonian-like deficits such as locomotor activity, motor coordination, anxiety and “depressive-like” behaviors. Then, we studied the impact of this intoxication on the neuronal activity, the globus pallidus (GP) and subthalamic nucleus (STN). At the end of experiments, post-mortem tissue level of the three monoamines (dopamine, norepinephrine and serotonin) has been determined. The experiments were carried out in adult Sprague-Dawley rats, daily treated with MnCl_2_ (10 mg/kg/, i.p.) for 5 weeks. We show that manganese progressively reduced locomotor activity as well as motor coordination in parallel with the manifestation of anxiety and “depressive-like” behaviors. Electrophysiological results show that, while majority of GP and STN neurons discharged regularly in controls, manganese increased the number of GP and STN neurons discharging irregularly and/or with bursts. Biochemical results show that manganese significantly decreased tissue levels of norepinephrine and serotonin with increased metabolism of dopamine in the striatum. Our data provide evidence that manganese intoxication is associated with impaired neurotransmission of monoaminergic systems, which is at the origin of changes in basal ganglia neuronal activity and the manifestation of motor and non-motor deficits similar to those observed in atypical Parkinsonism.

## Introduction

Manganese (Mn) is an essential trace element that is naturally present in water, air, soil and food. It plays an important role in the development and functioning of the brain as it is a co-factor for several enzymes involved in neurotransmitter synthesis and metabolism [1], [2]. While it is essential at physiological levels, excessive accumulation of Mn in the brain may cause serious central nervous system dysfunctions known as manganism, an extrapyramidal motor disorder similar to Parkinsonism [3]–[5]. In addition to motor disturbances, cognitive as well as neuropsychiatric deficits have been reported [6], [7]. Although the underlying mechanisms of Mn-induced neurotoxicity are still not known, neuroimaging studies indicated that manganism is associated with Mn accumulation in the basal ganglia, including the globus pallidus (GP) and subthalamic nucleus (STN) [8]–[11], two basal ganglia nuclei involved in the control of motor and non-motor functions. Several experimental animal studies have shown that Mn alters dopaminergic neurotransmission (see [12] for review) however, contrasting data are reported. While some studies have shown a decrease in dopamine (DA) neurotransmission [13]–[16], others have reported an increase [17], [18], both increase and decrease [19] or no change [20]–[22] in the tissue level of DA in Mn intoxicated animals. In addition to DA, other neurotransmitters such as norepinephrine (NE) and serotonin (5-HT) may be altered by Mn exposure. In fact a decrease in tissue levels of NE and 5-HT has been reported in non-human primates exposed to Mn [23], [24]. Together, these studies suggested that Mn alters the integrity of monoaminergic neurotransmission. However, its impact on basal ganglia neuronal activity remains to be investigated. We hypothesized that Mn exposure could induce defective monoaminergic neurotransmission resulting in a disorganization of the neuronal activity of GP and STN, which might be at the origin of motor and non-motor deficits.

The present study aimed to investigate the effects of Mn exposure upon (i) motor and non-motor functions including locomotor activity, motor coordination, anxiety, anhedonia and “depressive-like” behaviors, and on (ii) the neuronal activity of GP and STN. Post-mortem high performance liquid chromatography (HPLC) was used to control the potential change in tissue contents of DA, NE and 5-HT.

## Materials and Methods

### Ethics Statement

All animal experiments were carried out in strict accordance with the Council Directive 2010/63EU of the European Parliament and the Council of 22 September 2010 on the protection of animals used for scientific purposes. The protocol was approved by the Committee on the Ethics of Animal Experiments “*Comité d’éthique pour l’expérimentation animale Bordeaux”* (Permit Number: 50120125-A).

### Animals and Mn Administration

**Figure 1:**
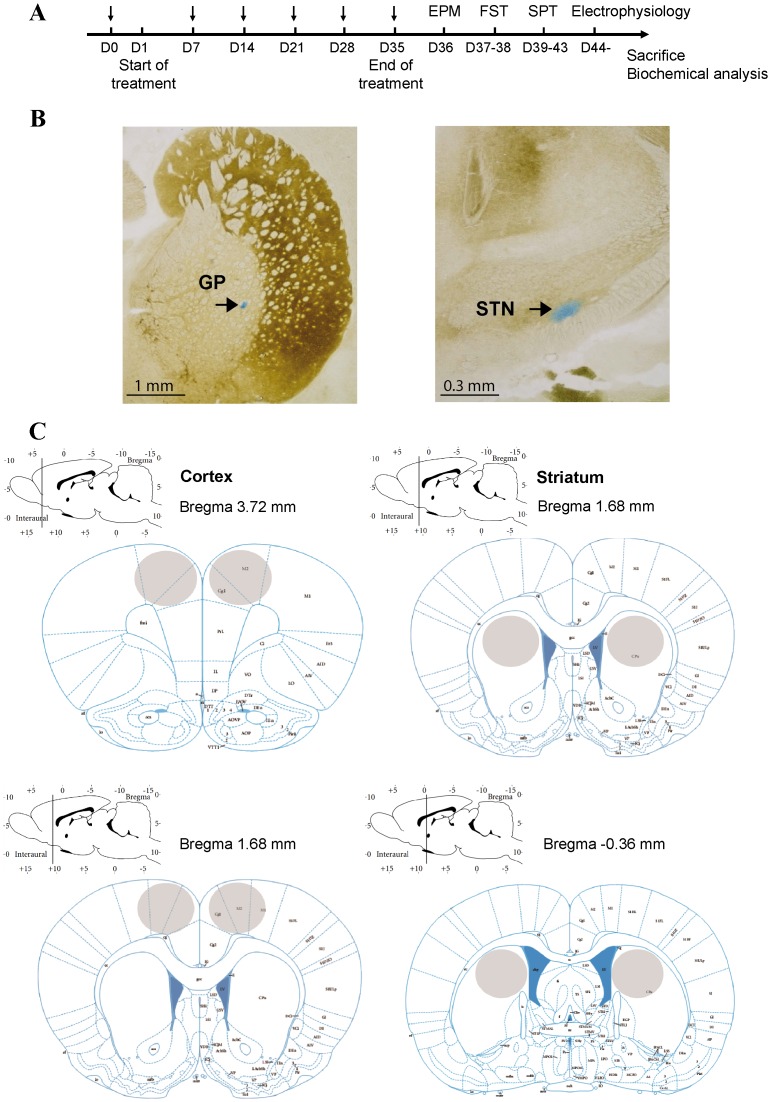
Experimental design and histological verifications. (**A**) Schematic presentation of the experimental design with a time course of all the behavioral tests (EPM: elevated plus maze, FST: forced swim test, SPT: sucrose preference test) and electrophysiological recordings. The vertical arrows correspond to the days when the rats were submitted to the open field and rotarod tests. (**B**) Histological sections showing the location of the pontamine sky blue dots in the GP (left) and the STN (right). (**C**) Location of brain regions sampled for biochemistry studies. Circles delineate the rostral (top) and caudal (bottom) limits of the sampled regions of cortex and striatum used for HPLC.

Male Sprague-Dawley rats (Centre d’Elevage Depré, Saint-Doulchard, France) weighing approximately 300 g at the beginning of the experiment were used in the present study. Animals arrived at least one week before use, and were housed in a room maintained under constant temperature (24°C) and humidity conditions (45%) with a 12∶12 h light–dark cycle (light on at 7∶00 AM). Food and water were available *ad libitum*. The rats were randomly assigned to 2 separated groups: intoxicated animal group (n = 12) received daily intraperitoneal injections of manganese chloride tetrahydrate (MnCl_2_.4H_2_O, Sigma-Aldrich, St. Louis, MO, USA) dissolved in saline (NaCl, 0.9%) at the dose of 10 mg/Kg for 5 weeks, and the control animal group (n = 11) received equal volume of saline in the same conditions. The dose of Mn was chosen based on several literature data showing a significant increase in Mn accumulation in brain tissues and alteration of the biochemical properties in the rat [13], [25]–[27]. The route of administration and the chemical form of Mn used were also chosen on the basis of the study of Roels et al. [28]. The animal’s weight was monitored throughout the experiment and the volume of Mn solution was adjusted every day taking into account the weight variation. In a first set of experiments, Mn treated rats (n = 7) and their controls (n = 5) were submitted to the open field, then they were used for electrophysiological recordings of STN neurons. In a second set of experiments, Mn treated rats (n = 5) and their controls (n = 6) were first submitted to the open field before they were used for non-motor tests and electrophysiological recordings of GP neurons. As the results of the open field parameters obtained in the two sets of experiments were not significantly different, they were pooled. The Figure 1A summarizes the experimental design, with a time course of all the motor and non-motor behavioral tests. After the last behavioral test, animals were used for electrophysiological recordings of GP and STN neurons. Immediately after each electrophysiological session, the rats were sacrificed and the brains removed for postmortem biochemical and histological assessments.

### Assessment of Exploratory and Locomotor Activities (Open Field)

Spontaneous horizontal and vertical (rearing) locomotor activities and stereotyped movements (grooming, washing, and other movements with low amplitudes and independent of locomotor activity) were measured using a photoelectric actimeter (Actitrack, Panlab, Barcelona, Spain), as previously described [29]. The actimeter consisted of a transparent platform (45×45 cm) equipped with photoelectric cells. Light beams detected movement, and the total locomotor activity of each rat was recorded in two consecutive sessions of 10 minutes, the two sessions of 10 min were used for data analysis. The first session was used to evaluate exploratory activity, and the second session evaluated the motor behavior of the animals. All testing in the actimeter were done in an isolated room. After 3 days of habituation, the locomotor activity was assessed at different time points, before the beginning of treatment (at day 0: D0, Fig. 1A) and during all treatment period once a week.

### Assessment of Motor Coordination (rotarod)

The effect of Mn on motor coordination was investigated by training the rats to remain on a rotarod (Bioseb, *in vivo* Research Instruments, Spain), as previously described [30], [31]. During the training period of 3 days, each rat was placed on a horizontal rod (7 cm diameter) rotating at a gradually increasing speed from 4 to 20 rotations per minute (rpm) for a maximum of 15 min by which time a steady baseline level of performance was reached. The day after training (D0) and once a week during all treatment period, the motor coordination was recorded for each animal during three trials. The latency to fall off the rotarod was recorded and the time limit was fixed at 3 minutes. The mean time of three trials was used for analysis.

### Assessment of Anxiety

Animals were tested in the elevated plus maze to assess anxiety-related behavior, as previously reported [32]. The elevated plus maze consisted of two open arms (50 cm long×10 cm wide) and two closed arms (50 cm long×10 cm wide×38.5 cm high), with an open roof arranged around a central platform (10×10 cm^2^). Arms of the maze form a cross, with two open arms being opposite each other. A camera was mounted 1.5 m above the elevated plus maze to record the frequency and duration of open and closed arm visits under red light. Animals were placed onto the central platform, facing one of the open arms. Each animal was allowed to explore the maze for 5 min. The time spent in the open and closed arms as well as the number of entries into the arms were considered when the animal entered an arm with all four paws. Animals were considered anxious when they enter less and spent less time in the open arms.

### Assessment of Anhedonia and “Depression-like” Behavior

#### Sucrose preference test

The Sucrose preference test was used, as previously described [33], [34]. Rats were placed in individual cages with food and water *ad libitum*. Animals were first habituated for 72 h with the presence of two water bottles and their position was randomly changed as many times as possible to prevent a place preference. The next 2 days when the light turns off at 7∶00 PM, pre-weighed water and 1% sucrose-containing bottles were placed on the home cage and rats were allowed to drink for 2 h. During these 2 h, the position of the bottles was changed four times. Water and sucrose absorption was measured by weighing the bottles before and after the test. Sucrose consumption was calculated as follows: 100× [sucrose intake (g)/(sucrose intake (g) + water intake (g))].

#### Forced swim test

The forced swim test was conducted as previously described [35]. Each rat was placed individually in a transparent plexiglass cylinder (diameter: 20 cm, height: 50 cm) containing water at 23±2°C. The water was 30 cm deep to prevent that the rats touch the bottom of the cylinder with their paws or the tail. The first day of habituation, the rats were forced to swim for 15 min; 24 h after, on the test day three categories of behavioral activity (climbing, swimming and immobility) were recorded during the 5 min test session. Off line analyses were performed as previously reported [36]. A rat was judged to be immobile when he remained floating in the water without struggling and was making only those movements necessary to keep its head above water. After the forced swim test, the rats were dried with a towel and placed under a warming lamp, then returned to their home cage.

### Extracellular Single Unit Recordings

Extracellular single-unit recordings were made in control and intoxicated rats under urethane anesthesia (1.2 g/kg i.p.) as previously reported [37], [38]. A single glass micropipette electrode (impedance: 8–12 MΩ; aperture 0.5 µm) was filled with 4% pontamine sky blue in 3 M NaCl and then lowered into the GP or STN according to the stereotaxic coordinates of the rat brain atlas of Paxinos and Watson [39] (in mm relative to bregma, AP: −0.9, L: −3, D: 4.5–7.5 for GP and AP: −3.8, L: −2.5, D: 6.8–8.2 for STN). Extracellular neuronal activity was amplified, band-pass filtered (300–3000 Hz) using a preamplifier (Neurolog, Digitimer, UK) and transferred via a Powerlab interface (AD Instruments, Charlotte, NC, USA) to a computer equipped with Chart 5 software (AD Instruments). Only neuronal activity with a signal-to-noise ratio >3∶1 was recorded and used for further investigation. Basal firing of neurons was recorded for 10 min, and at the end of each session the recording site was marked by electrophoretic injection (Iso DAM80, World Precision Instruments, Hertfordshire, UK) of pontamine sky blue after recording the last neuron of the last trajectory through the micropipette at a negative current of 20 µA for 10 min. After completion of the experiments, animals were sacrificed, the brains removed, frozen in isopentane at −45°C and stored at −80°C. Fresh-frozen brains were cryostat cut into 20 µm coronal sections and acetylcholine esterase staining was used as previously described [29] to determine the location of the pontamine sky blue dots marking the recording site in the recorded structure. Only brains with clear blue dots in the GP and STN (Figure 1B) were used for data analysis.

#### Data analysis

The activity of each neuron was analyzed with a spike discriminator using a spike histogram program (AD Instruments), and firing parameters were calculated using Neuroexplorer program (Alpha Omega, Nazareth, Israel). Firing rates were expressed as the averaged frequency of discharge calculated over the 10 min period of stabilization, and the value for each group is the mean ± SEM. Firing patterns were analyzed as previously described [40] using the method of Kaneoke and Vitek [41]. Three patterns were determined: a regular pattern, with a discharge density distribution of spike train that follows a near-normal distribution; an irregular pattern, which follows a Poisson distribution; and a bursty pattern, with a discharge density histogram that follows two different distributions. The number of cells discharging in each pattern was expressed as a percentage of the total number of neurons recorded.

### Biochemical Assessment of Monoamines

Tissue contents of DA and 5-HT together with their respective metabolites (DOPAC, HVA and 5-HIAA) were measured in the striatum and the frontal cortex. These structures were chosen for three main reasons. First, cortex and striatum are classically used to inform on the biochemical status of monoaminergic fibers because they receive, as the other basal ganglia nuclei, DAergic, NEergic and 5-HT afferents from the *pars compacta* of substantia nigra, locus coeruleus and dorsal raphe respectively. Second, these structures are easier than others to punch in a reproducible manner according to the coordinates of the rat brain atlas of Paxinos and Watson [39] (Fig. 1C). Third, there is a marked distinction of the neurochemical profile between the two structures [32], [42], so that we can ensure that the dissected structures correspond to enriched striatum and frontal cortex. Monoamines tissue concentrations were measured by HPLC with electrochemical detection in these brain areas of Mn-intoxicated rats and controls, as previously described [32], [43]. Dissected striatum and frontal cortex were punched at −20°C, and stored at −80°C until their use in biochemical assays. The tissues were homogenized in 100 µl of 0.1 N HClO_4_ and centrifuged at 13,000 rpm for 30 min at 4°C. Aliquots of the supernatants were diluted in the mobile phase (1/4 for the striatum) and injected into the HPLC column (Chromasyl C8, 150×4.6 mm, 5 µm) protected by a Brownlee-Newgard Precolumn (RP-8, 15×3.2 mm, 7 µm) (CIL, Sainte-Foy la Grande, France). The mobile phase delivered at 1.2 ml/min; flow rate was as follows (in mM): 60 NaH2PO4, 0.1 disodium EDTA, and 2 octane sulfonic acid plus 7% methanol, adjusted to pH 3.9 with orthophosphoric acid and filtered through a 0.22 µm Millipore filter. Detection of compounds was performed with a coulometer detector (CoulochemII, ESA, Paris, France) coupled to a dual electrode analytic cell (model 5011, ESA). The potential of the electrodes was set at +350 and −270 mV. Results are expressed as ng/g of tissue, and each value is the mean ± SEM.

### Statistical Analysis

Statistical analyses were performed using Sigmaplot (Systat Software, San Jose, USA). The time course of body weight of the animals and motor behavior parameters, in the two groups were compared using a two way ANOVA with repeated measures (day and group) with Holm-Sidak *post hoc* analysis as a multiple comparison procedure. The parameters of non-motor behaviors as well as biochemical results were compared using a Mann-Whitney non-parametric *t*-test. For electrophysiological analysis, changes in the proportion of different firing patterns (regular, irregular and bursty) were determined using a Chi-square test. Firing rates from Mn-treated rats and controls were compared using a Student *t*-test. A *P*-value <0.05 was considered as significant.

## Results

### Body Weight Changes during Manganese Treatment

**Figure 2:**
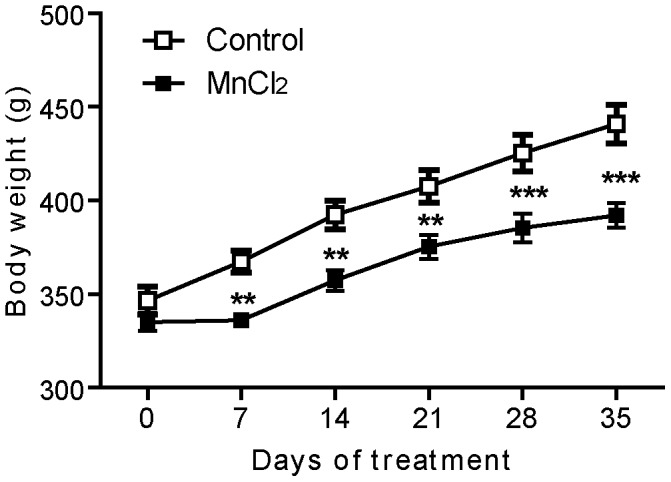
Effect of manganese on body weight gain. Values are the mean ± SEM. Data from Manganese-treated rats (*n* = 12) and controls (*n* = 11) were compared using the two way repeated measures ANOVA and Holm-Sidak *post hoc*. **p<0.01, ***p<0.001.

Body weight of animals was measured before and during the period of treatment. The evolution of body weight of Mn-treated animals was significantly different from that of control animals (Two way ANOVA RM, *post hoc* Holm Sidak method, F = 5.22, *p = 0.0003*, Fig. 2). Before starting Mn-exposure (D0) the animals of both groups had a nearly same body weight with a mean ± S.E.M. of 335.2±4.7 g in Mn-treated rats and 340.7±4.3 g in controls. During the period of intoxication, while control animals showed a regular progressive increase in their body weight, Mn intoxicated animals showed a slow increase in weight.

### Mn Treatment Reduced Exploratory Activity

**Figure 3:**
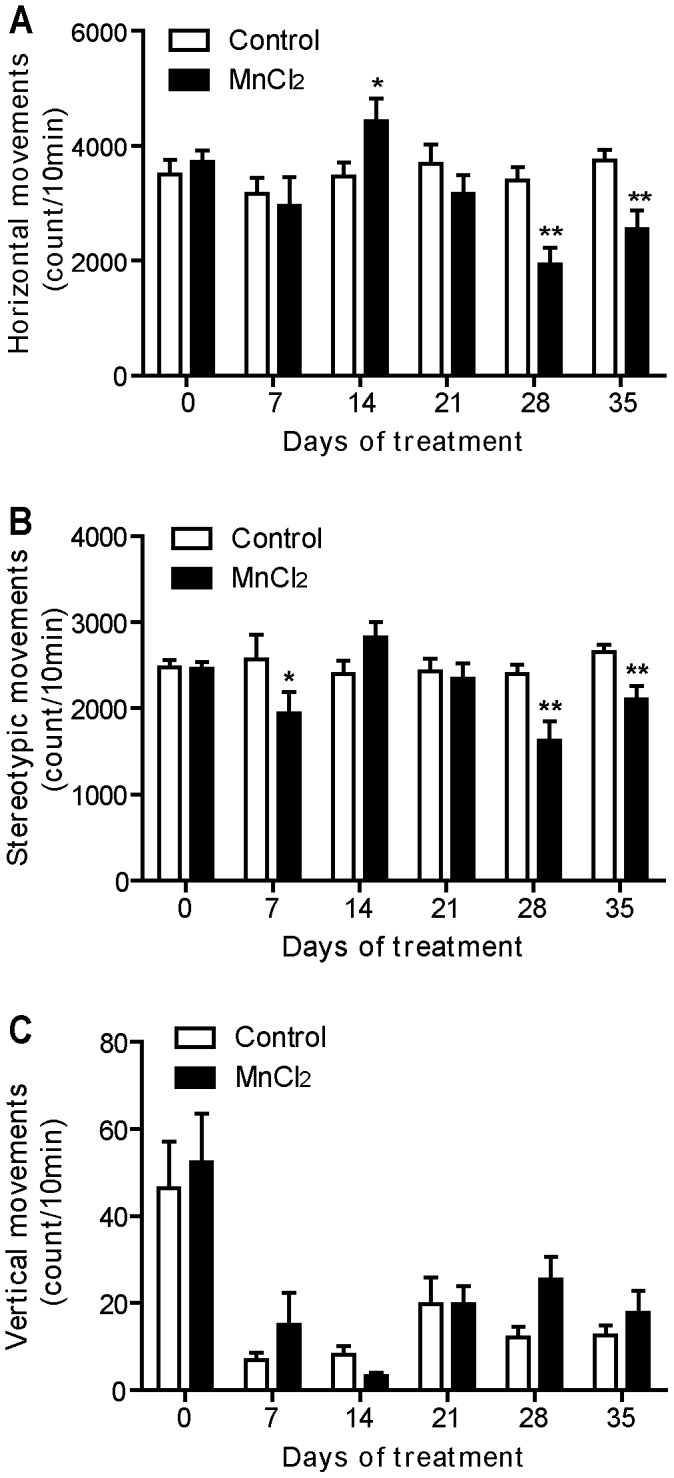
Manganese progressively reduced exploratory activity. Exploratory activity histograms represent the number of horizontal (A), stereotypic (B), and vertical movements (C) recorded during the first session of 10 min in the “open field” actimeter before and during all period of treatment. Values are the mean ± SEM. Data from manganese-treated rats (*n* = 12) and controls (*n* = 11) were compared using the two way repeated measures ANOVA and Holm-Sidak *post hoc*. *p<0.05, **p<0.01.

Horizontal movements, rearing, and stereotypic movements measured during the first 10 min session in the open field actimeter correspond to the exploratory activity of the animals. As shown in the figure 3, Mn intoxication affected horizontal and stereotypic movements in comparison with control animals (two way ANOVA RM, *post hoc* Holm Sidak method, F = 5.02, *p*<0.001, Fig. 3A and F = 3.95, *p* = 0.003, Fig. 3B, respectively). Mn intoxication significantly decreased horizontal exploratory activity on days 28 (*p*<0.01) and 35 (*p*<0.01), with a transient increase at day 14 (*p*<0.05, Fig. 3A). The decrease was also observed in stereotypic movements on days 28 (*p*<0.01) and 35 (*p*<0.01, Fig. 3B). However, no significant change was observed in the vertical activity between Mn-intoxicated and control animals (F = 0.70, *p* = 0.62, Fig. 3C).

### Mn Treatment Reduced Locomotor Activity

**Figure 4:**
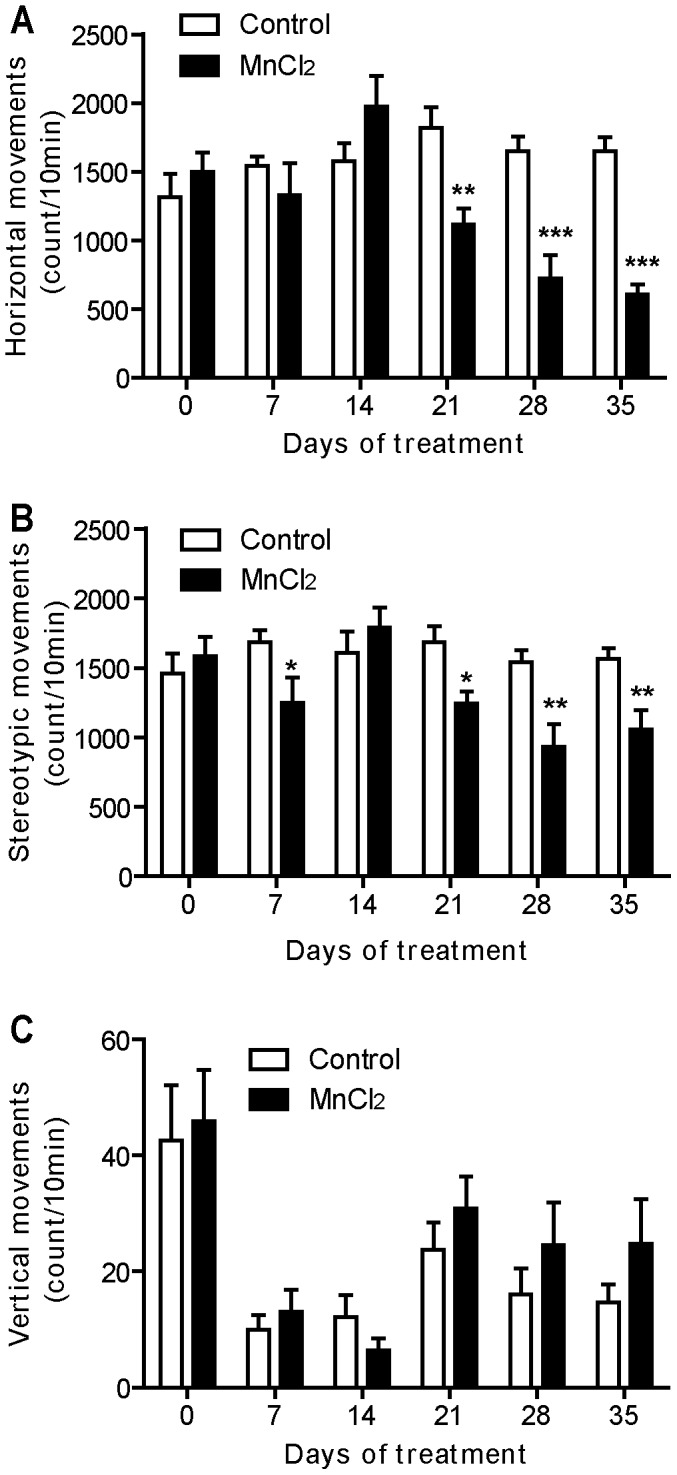
Manganese progressively reduced locomotor activity. Locomotor activity histograms represent the number of horizontal (A), stereotypic (B), and vertical movements (C) recorded during the second 10 min session in the “open field” actimeter before and during all the period of treatment. Values are the mean ± SEM. Data from manganese-treated rats (*n* = 12) and controls (*n* = 11) were compared using the two way repeated measures ANOVA and Holm-Sidak *post hoc*. *p<0.05, **p<0.01, ***p<0.001.

Results of the second session of 10 min in the actimeter showed that Mn intoxication progressively and significantly affected the scores of horizontal and stereotypic movements over time compared to control animals (two way ANOVA RM, *post hoc* Holm Sidak method, F = 9.950, *p*<0.001 Fig. 4A and F = 3.90, *p* = 0.003 Fig. 4B respectively). Mn intoxication significantly decreased horizontal locomotor activity on days 21 (*p*<0.01), 28 (*p*<0.001) and 35 (*p*<0.001) (Fig. 4A). The decrease was also observed in stereotypic movements on days 7 (*p*<0.05), 21 (*p*<0.05), 28 (*p*<0.01) and 35 (*p*<0.01) (Fig. 4B). However, no change was observed in the vertical locomotor activity (F = 0.47, *p* = 0.79, Fig. 4C).

### Mn Treatment Reduced Motor Coordination

**Figure 5:**
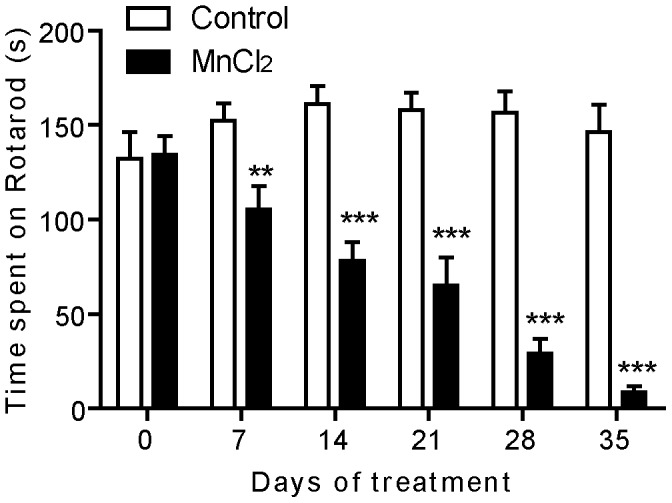
Manganese progressively reduced motor coordination. Motor coordination histogram represents the time that rat stay on the bar of rotarod before and during all period of treatment. Values are the mean ± SEM. Data from manganese-treated rats (*n* = 5) and controls (*n* = 5) were compared using the two way repeated measures ANOVA and Holm-Sidak *post hoc*. **p<0.01, ***p<0.001.

Compared to controls, Mn intoxication significantly changed the time spent on the rotarod bar (Two way ANOVA RM, *post hoc* Holm Sidak method, F = 21.34, *p*<0.001, Fig. 5). A dramatic progression in the decrease of time spent on the rotarod was observed, starting at low level on day 7 (*p*<0.01) to reach a severe level on day 35 (*p*<0.001).

### Mn Treatment Reduced the Time Spent in Open Arms of the EPM

**Figure 6:**
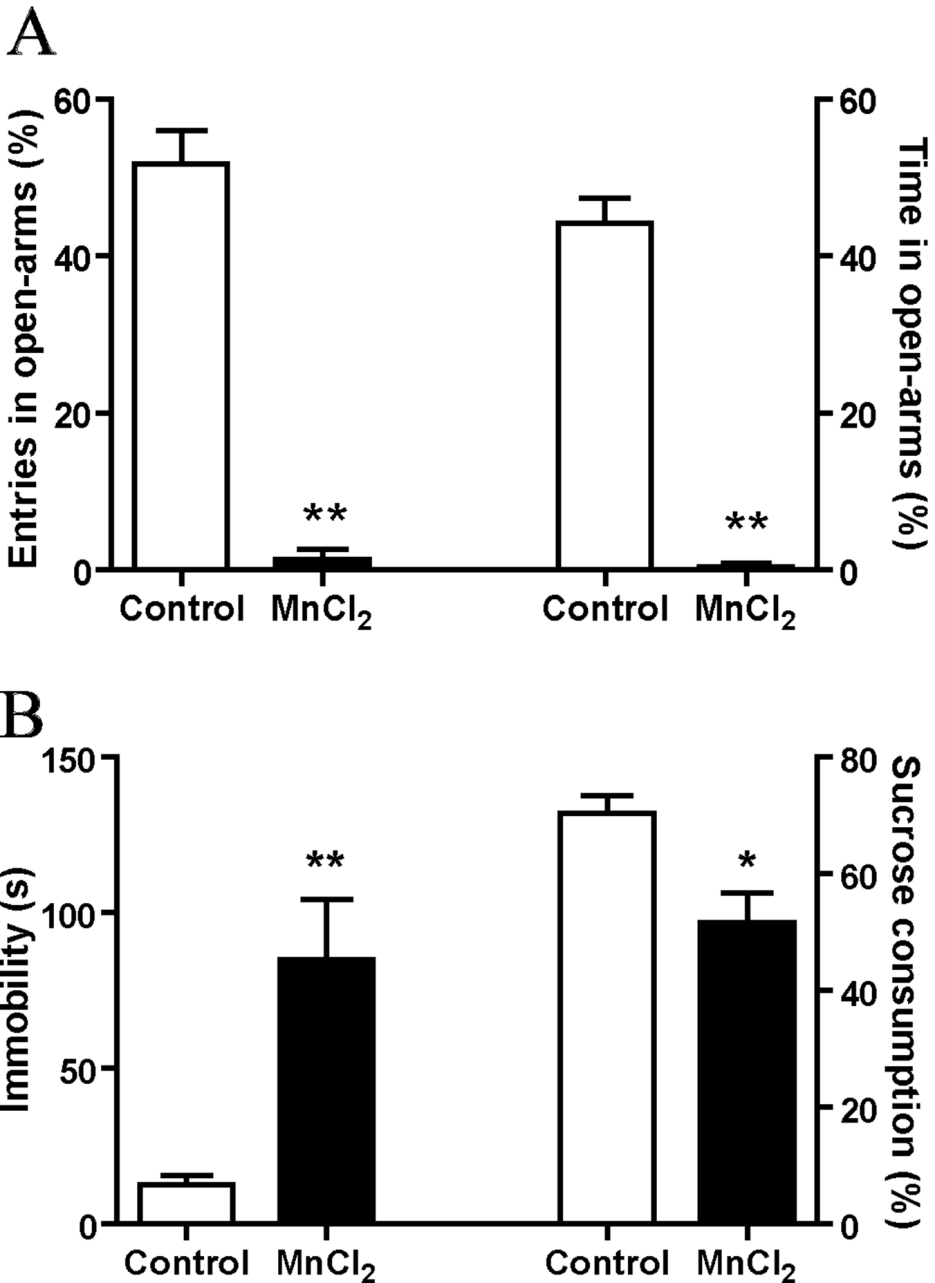
Manganese induced anxiety, anhedonia and depressive like behaviors. (A) Histograms showing the percentage of the number of entries into the open arms of elevated plus maze relative to the total number of entries into the four arms (left) and the percentage of time spent in the open arms relative to the total time spent in the four arms (right). (B) Histograms showing the immobility duration in the forced swim test and the percentage of sucrose consumption relative to the total consumption. Values are the mean ± SEM. Data from Manganese-treated rats (n = 5) and controls (n = 6) were compared using the Mann–Whitney test. *p<0.05, **p<0.01.

Anxiety behavior was assessed using the EPM at the end of Mn intoxication as this test cannot be repeated. Compared to control animals, Mn treatment dramatically decreased the number of entries (1.34±1.34 versus 51.78±4.146 in control rats, Mann-Whitney test, *p*<0.01) and time spent in open arms (0.46±0.46 sec versus 44.17±3.23 sec in control rats, Mann-Whitney test, *p*<0.01, Fig. 6A).

### Mn Treatment Increased “Depressive-like” Behavior

Depressive-like behavior was assessed using the forced swim test. Mn treatment significantly increased the immobility time compared to control animals (85.00±19.49 sec versus 12.50±3.35 sec in control rats, Mann-Whitney test, *p*<0.01, Fig. 6B). Anhedonia was assessed using the sucrose preference test. As shown in figure 6B, Mn treatment significantly reduced the sucrose consumption compared to control animals (51.65±5.19% versus 70.39±3.06% in control rats, Mann-Whitney test, *p*<0.05).

### Effects of Manganese on GP and STN Neuronal Activity

**Figure 7:**
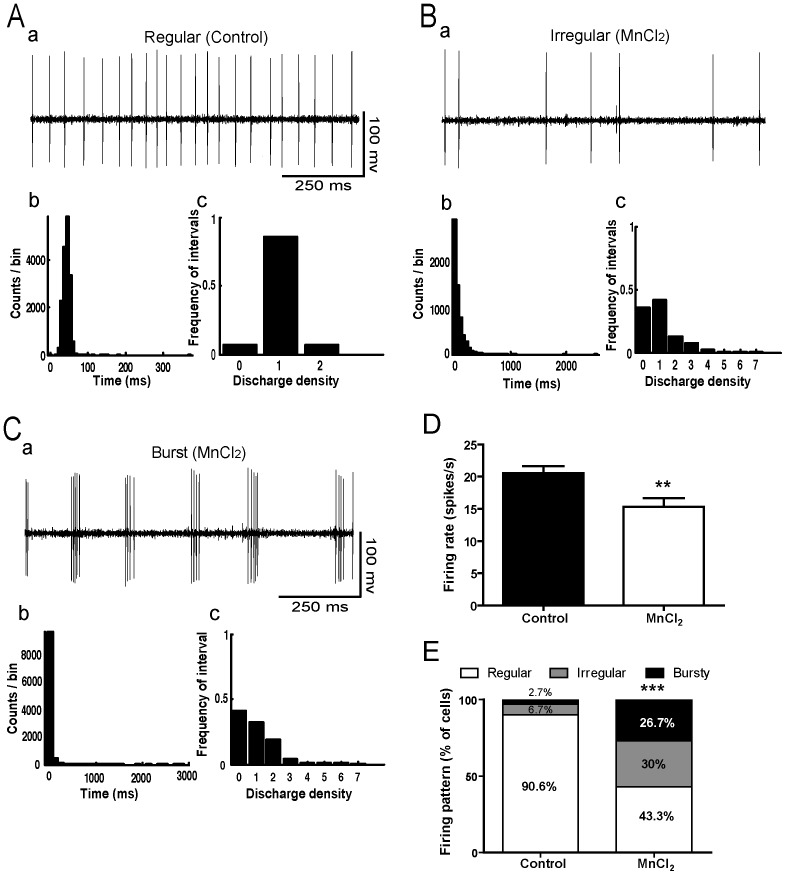
Manganese decreased the firing rate of GP neurons and increased the proportion of bursty and irregular neurons. (ABC) Representative examples of spike trains recorded in the GP (a) with interspike interval histogram (b) and density histogram (c), showing a regular pattern in control rats (A) and irregular and bursty patterns in manganese (Mn)-treated animals (B and C respectively). (D) Firing rate histograms with values as the mean ± SEM. Firing rate data from manganese-treated rats and controls were compared using Student *t*-test. **p<0.01. (E) Firing pattern histograms showing the proportion of GP cells discharging regularly, irregularly or with bursts. Changes in the proportion of different firing patterns were analyzed using a chi square test. ***p<0.001 in comparison with controls.

**Figure 8:**
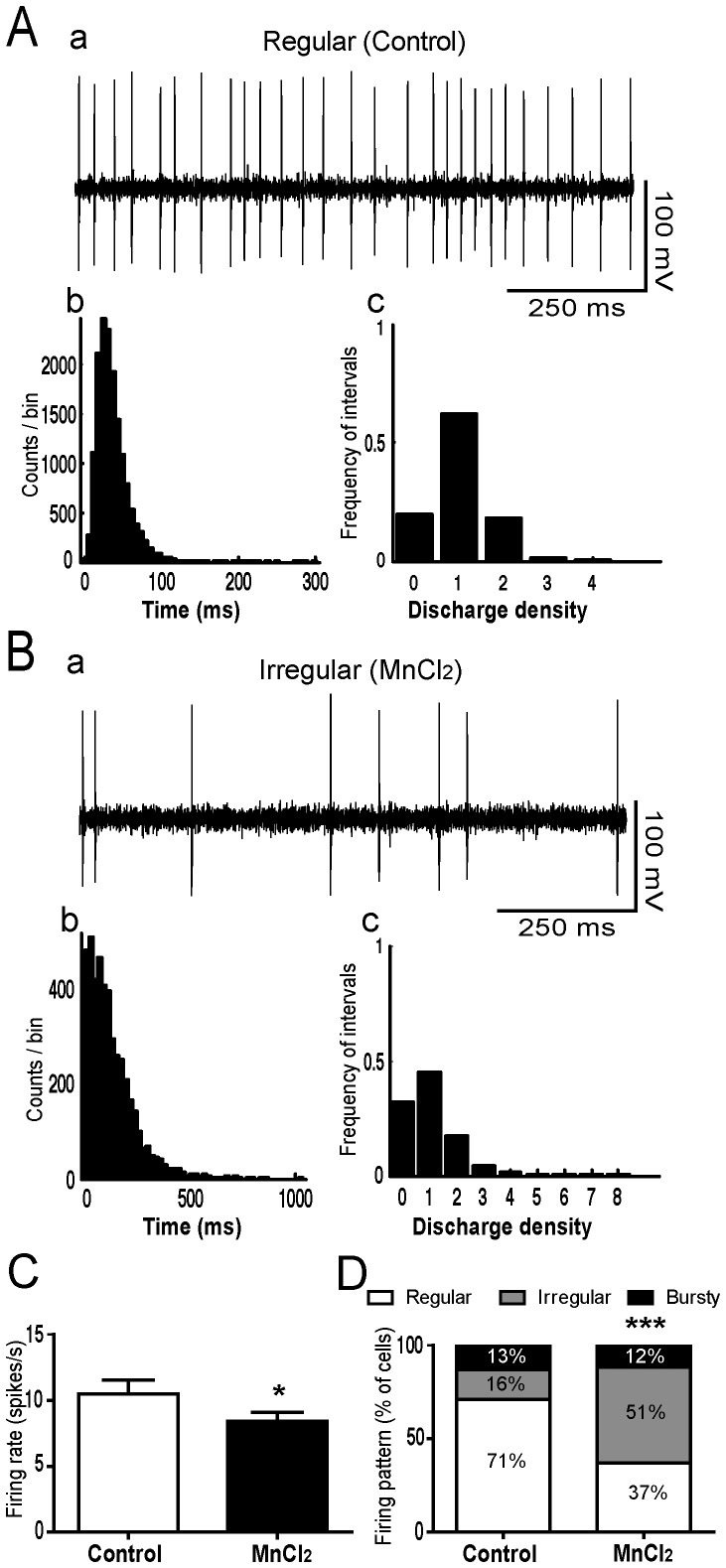
Manganese decreased the firing rate of STN neurons and increased the proportion of irregular neurons. (AB) Representative examples of spike trains recorded in the STN (a) with interspike interval histogram (b) and density histogram (c), showing a regular pattern in control rats (A) and irregular pattern in manganese (Mn)-treated animals (B). (**C**) Firing rate histograms with values as the mean ± SEM. Firing rate data from manganese-treated rats and controls were compared using Student *t*-test. (D) Firing pattern histograms showing the proportion of STN cells discharging regularly, irregularly or with bursts. Changes in the proportion of different firing patterns were analyzed using a chi square test. *p<0.05, ***p<0.001 in comparison with controls.

In control rats (n = 6), the mean firing rate of GP neurons (n = 75) was 20.53±1.13 spikes/s. Majority of these neurons discharged regularly (90.6%) (Fig. 7) and only few discharged irregularly (6.7%) or with bursts (2.7%). In Mn intoxicated rats (n = 5), the mean firing rate of GP neurons (n = 60) significantly decreased to 15.35±1.31 spikes/s (Student *t-test*, *p*<0.01, Fig. 7). Moreover, Mn significantly reduced the percentage of GP neurons discharging regularly (43.3%) and increased the percentage of cells discharging both irregularly (30%) and in bursts (26.7%) (χ^2^ = 35.78, *p* = 0.002, Fig. 7). Likewise, the firing rate of STN neurons significantly decreased from 10.4±1 spikes/s (n = 59) in control animals (n = 5) to 8.4±0.7 spikes/s (n = 71) in Mn intoxicated animals (n = 7) (Student *t*-test, *p*<0.05, Fig. 8). Concerning the firing pattern, Mn intoxication significantly decreased the proportion of STN neurons discharging regularly (37% versus 71% in control animals) and increased the percentage of cells discharging irregularly (51% versus 16% in control animals) (χ^2^ = 19.078, *p* = 0.0001, Fig. 8).

### Mn Affected Monoamines Tissue Levels

Table 1 summarizes the tissue content of DA and its metabolites (DOPAC and HVA), 5-HT and its metabolite 5-HIAA in the striatum, as well as NE, 5-HT and 5-HIAA in the frontal cortex. In the striatum, Mn significantly increased the tissue content of DA by 33.84% (Student *t*-test, *p*<0.05) and its primary metabolite DOPAC by 46.91% (*p*<0.05) without affecting the level of the secondary metabolite HVA (*p* = 0.08). In this structure, 5-HT and 5-HIAA tissue levels were not affected (*p* = 0.33 and *p* = 0.93 respectively). In the frontal cortex, the tissue content of NE and 5-HT significantly decreased in Mn intoxicated rats when compared with controls (−17.58%, *p*<0.05 and −27.28%, *p*<0.05 respectively), however, the tissue content of 5-HIAA was not affected (*p* = 0.06).

**Table 1:** Biochemical analysis of manganese-induced changes in the tissue level of monoamines.

Striatum (ng/g of tissue)						Frontal cortex (ng/g of tissue)		
	DA	DOPAC	HVA	5-HT	5-HIAA	NE	5-HT	5-HIAA
**Control**	6249±481.8	872.6±73	441.7±31.2	197.1±28.3	359.5±38.6	133.1±9.5	146±11.7	243±18.4
**MnCl_2_**	**8364±968** *	**1282±152.3** *	673.1±107.6	168.6±28.2	381.8±59.4	**109.7±4.9** *	**106.6±13.4** *	191.5±22.9

Manganese-induced a decrease in the tissue level of NE and 5-HT in the frontal cortex and increase of DA metabolism in the striatum. Tissue contents of DA, DOPAC, HVA, 5-HT, and 5-HIAA in the striatum and NE, 5-HT, and 5-HIAA in the frontal cortex measured by HPLC in manganese-treated rats and their controls. Values are concentrations in ng/g of wet tissue presented as the mean ± SEM. Statistical analysis using Mann–Whitney test was performed;

*p<0.05 in comparison with control.

## Discussion

The present study shows for the first time that Mn intoxication induced motor and non-motor deficits paralleled by dramatic changes in the firing rate and patterns of GP and STN neurons and decreased tissue levels of norepinephrine and serotonin, in addition to an increase of dopamine metabolism. Our data suggest that the observed electrophysiological and biochemical changes might be at the origin of the motor and non-motor behavioral deficits induced by Mn.

We show that Mn intoxication induced a progressive slowing of body weight gain, in line with previous studies [26], [44]–[46]. This effect may reflect a broad interference with normal physiological processes, such as deficiency in energy metabolism and functional alterations in the hypothalamic nuclei contributing to the control of body weight [47], [48].

Exploratory as well as locomotor activities measured during the 35 days of Mn intoxication revealed a progressive decrease in horizontal and stereotypic movements compared to control animals. The observed decrease in spontaneous locomotor activity in the open field is consistent with earlier observations [26], [28], [49], [50]. In addition, Betharia and Maher [51] observed a decrease in locomotor activity in male rats pups, which were exposed to Mn during gestation and lactation. One possibility is that the locomotor activity may be influenced by the reduction of weight gain in Mn-intoxicated animals. However, it is unlikely that the decrease in locomotor activity can be due to reduced weight gain as it has been reported that undernourished as well as malnutrished rats, who showed the same profile of weight gain reduction, increased their locomotor activity in the open field [52]. Moreover, we show that Mn intoxication significantly and gradually affected motor coordination expressed by the very short time spent on rotating bar in the rotarod test, in line with another study carried out in Mn-intoxicated mice [53]. Our results clearly show a link between locomotor and motor coordination deficits and Mn intoxication suggesting that Mn affects the central structures involved in the control of motor behavior such as basal ganglia. From a behavioral point of view, motor disabilities induced by Mn are somehow similar to those observed in rat models of parkinsonism [29], [32], [37] and also in rats submitted to the intoxication with lead, which is another heavy metal known for its neurotoxic effects [31]. Motor coordination deficit can also be due, at least in part, to ataxia. Indeed, excess accumulation of Mn due to impaired transport or failure of hepatic detoxification mechanisms may result in ataxia implicating the cerebellum in addition to basal ganglia [54].

Interestingly, Mn-induced motor deficits are not related to DAergic depletion comparable to that reported in Parkinson’s disease or in animal models of the disease [32], [37], [55]. Indeed, Mn intoxication increased the striatal tissue level of dopamine and its principal metabolite DOPAC. Contrasting data have been reported concerning the impact of Mn on the DAergic system, depending on the route of administration and the dosage used. Our results are in line with those of Ingersoll et al. [56] who reported that Mn in the drinking water significantly increased the level of dopamine and DOPAC in the striatum. However, it has been shown that DA contents were decreased after intrathecal administration [56] or inhalation of Mn [25], [57]. Interestingly, it has been shown that at lower doses, Mn increased DA and its metabolite levels, while the opposite effect was seen at higher doses [58]. The dose used in our study is considered as a low dose [58]. Thus, it is possible that the increase in tissue levels of DA and DOPAC may reflect a local action of Mn, which may precede a dysfunction of DAergic neurons. Although we do have direct evidence for an alteration of the DAergic tone in the striatum, the moderate increase in DA metabolism combined with the impairments of motor functions suggests a decreased sensitivity of dopamino-receptive neurons to DA as it has been described for neuroleptics [59]. Additionally, we cannot exclude the possibility that Mn can directly cause neurotoxicity in substantia nigra *pars compacta*, which may account for the change of DA level in the striatum. The overall picture leads to consider the alteration of other neurotransmitter systems in Mn intoxicated animals.

The decrease in cortical NE contents in our study suggests that this neurotransmitter might be a plausible candidate for explaining Mn-induced motor deficits. It may reflect an alteration of NEergic neurons of the locus coeruleus by Mn that results in a reduction of NEergic cortical input. Although NE is rarely associated with motor impairment in neurodegenerative diseases, growing experimental evidence suggest that this neurotransmitter might be involved in abnormal motor behaviors (see [60] for review). Indeed, knockout mice (Dbh−/−) that lack NE exhibits a robust impairment in motor function and motor coordination that is specifically related to the depletion of NE [61]. Furthermore, we have shown that systemic administration of selective adrenoceptor agonists and antagonists were able to modulate locomotor activity in the rat [37] and that DSP-4 treatment-induced selective NE depletion induced motor deficits [32].

In addition to the motor deficits, we show that Mn intoxication induced anxiety, as shown by the strong reduction in the time spent and number of entries in the plus maze open arms, as well as anhedonia and “depressive-like” behaviors as shown by the reduction in sucrose preference and the increase of immobility time in the forced swim test respectively. It is unlikely that anxiety and depressive-like behavior observed in our study can be due to the motor deficit developed by the animals. Indeed, in a recent study using the same tests [32] we have shown that 6-OHDA or DSP-4 treatments, which induced hypolocomotor activity, did not induce anxiety and depressive-like behavior. These non-motor disorders were developed in animals with combined lesions of the monoaminergic systems, which did not potentiate motor deficits. Moreover, results of the sucrose preference test, which is independent of locomotor activity as the data were normalized, corroborate those of the forced swim test. The non-motor disabilities might be more specifically related to a reduction of both NE and 5-HT tissue contents. Accordingly, previous studies including ours reported that failures in these two monoaminergic neurotransmission systems contribute to the emergence of neuropsychiatric disorders, such as anxiety and “depressive-like” behaviors [32], [62] (see [60] for review). However, the link between behavioral changes and increased DA tissue content is difficult to explain. Nevertheless, as previous anatomical and electrophysiological studies reported the existence of reciprocal and functional relationships between the three monoaminergic systems [42], [63], [64], it is likely that the non-motor abnormalities observed in our study may result from the imbalance in neurotransmission of the three monoaminergic systems.

Mn induced motor and non-motor deficits were paralleled by a disorganization of the electrical activity of GP and STN neurons. Indeed, a shift from a regular discharge pattern to irregular and/or burst firing patterns was observed in both GP and STN. These two basal ganglia nuclei are known to play a key role in the proposed models of motor and non-motor circuits [65], [66]. They are organized in five parallel cortico-basal ganglia-thalamo-cortical circuits, including the sensorimotor, associative and limbic circuits. GP and STN are the two principal components of the indirect pathway in these circuits, with the STN considered as a driving force of the output structures of basal ganglia. Based on our recent data [67], it is likely that the changes recorded in GP after Mn intoxication are a consequence of 5-HT depletion, but not NE depletion. Indeed, selective depletion of 5-HT by pCPA administration resulted in the same decrease of the firing rate and increase in the proportion of irregular and bursty neurons in GP [67]. However, DSP-4, which is selective for NE depletion did not alter by itself the firing rate or the pattern of GP neurons [67]. Interestingly, and in contrast to GP, the changes observed in STN neurons after repeated Mn intoxication are likely to be due to NE depletion, but not to 5-HT depletion. Indeed, DSP-4 administration resulted in the same decrease of the firing rate and increase in the proportion of irregular neurons recorded in the STN [32]. However, selective 5-HT depletion did not alter the firing rate nor the pattern of STN neurons [67]. Besides, lead intoxication, which reduced the tissue contents of NE without affecting the 5-HT system, changed in a similar way the firing activity of STN neurons [31]. In addition to the depletion of NE and 5-HT, we cannot exclude that the changes in the firing activity of GP and STN neurons can also be due to the accumulation of Mn in GP and STN. Neuroimaging studies have shown an accumulation of Mn in the basal ganglia in addition to the frontal and parietal cortex (see [68] for review).

From a pathophysiological point of view, the firing activity changes of GP neurons after Mn intoxication is nearly similar to those observed in the 6-OHDA rat model of Parkinson’s disease [69]–[72], although the origin is not the same. However, the changes in STN neuronal activity in Mn-intoxicated animals are different from those observed after 6-OHDA-induced DA depletion in the striatum. While 6-OHDA lesion induced a switch from regular to bursty pattern with oscillations [32], [38], [67], [70], Mn intoxication induced a switch from regular to irregular pattern (the present study). Together, these findings provide a logical explanation to the evidence that Mn-induced motor and non-motor deficits are not improved by L-Dopa therapy as it does in Parkinson’s disease (see [68] for review).

In conclusion, the present study provides evidence that manganese intoxication is associated with impaired neurotransmission of monoaminergic systems, which is at the origin of changes in basal ganglia neuronal activity and the manifestation of motor and non-motor deficits similar to those observed in atypical Parkinsonism.
